# Primary squamous cell carcinoma of the ovary. Review of the literature

**DOI:** 10.3389/fonc.2022.962613

**Published:** 2022-09-14

**Authors:** Yan Luo, Ce Bian

**Affiliations:** Department of Gynecology and Obstetrics, Key Laboratory of Birth Defects and Related Diseases of Women and Children, Ministry of Education, West China Second University Hospital, Sichuan University, Chengdu, China

**Keywords:** pure, primary, ovarian, squamous cell carcinoma, case report

## Abstract

Pure primary ovarian squamous cell carcinoma (oSCC) is very rare, with about 42 cases have been reported in the literature. Lacking effective treatment guidelines, the prognosis of oSCC is extremely poor. Here, we report a 52-year-old postmenopausal woman diagnosed with pure primary oSCC in our center. The patient received debulking surgery followed by chemotherapy with carboplatin, paclitaxel, and bevacizumab. The patient survived 11 months after surgery and died of tumor progression and multiple organ failure. We also present a review of the literature.

## Introduction

Ovarian squamous cell carcinoma (oSCC) accounts for 1% of ovarian cancer (1). Most of these tumors arise from malignant transformation of mature cystic teratoma, and others are associated with endometriosis or Brenner tumor, and rare metastasis from other organs SCC. Pure primary oSCC that occurs in ovary without any pre-existing ovarian lesion is extremely rare, and most were described in case reports. Compared with other histologic ovarian tumors, the prognosis of oSCC is very poor, and its effective treatment has not been established (1–14). Here, we report a case of pure primary oSCC underwent optimal debulking surgery combined with systemic chemotherapy. However, the tumor rapidly metastasized to the lateral wall of the pelvis and the rectum during chemotherapy, and the patient died of multiple organ failure 11 months later since debulking surgery.

## Case presentation

**Figure 1:**
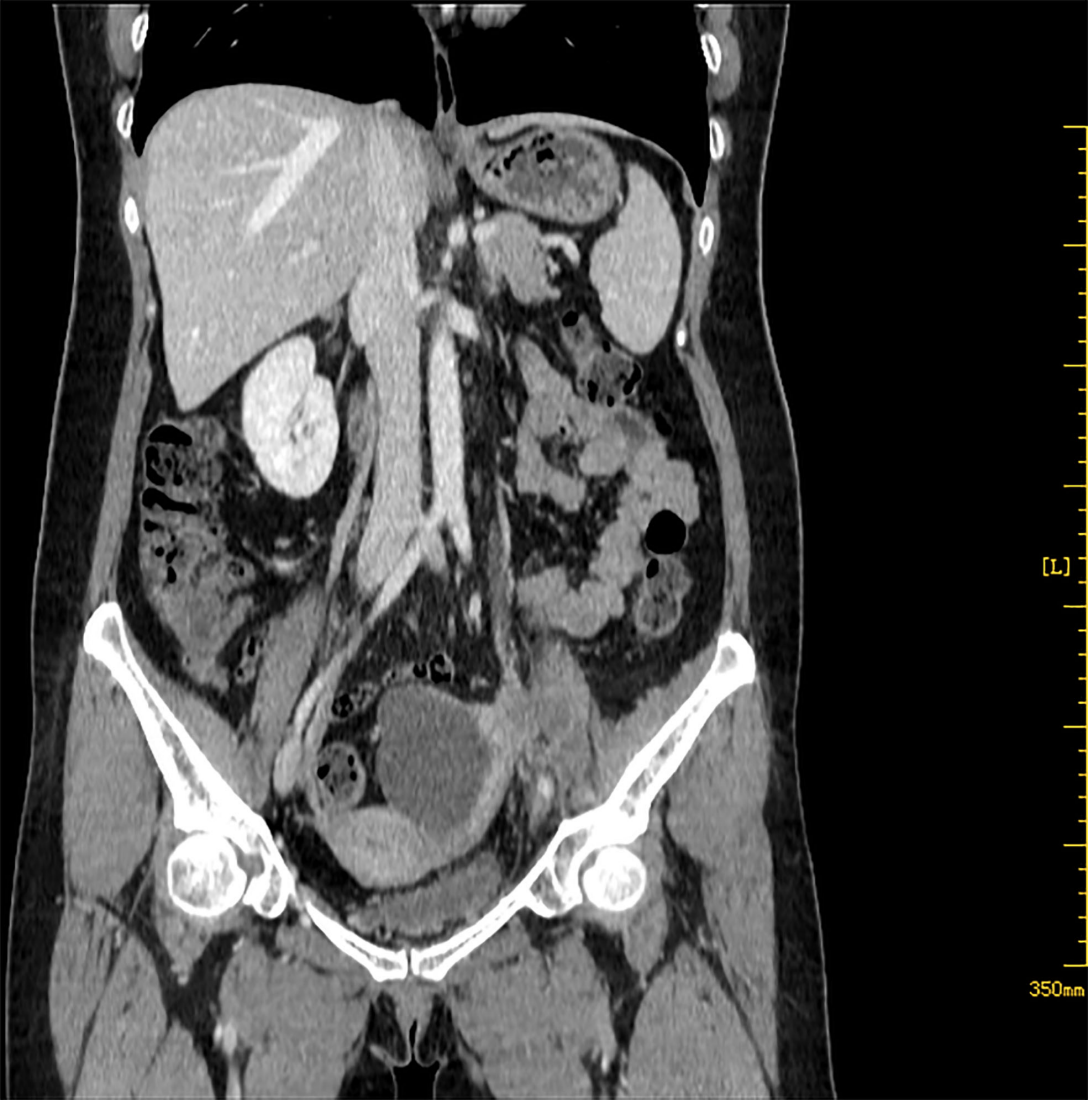
Abdominal CT shows a 7.1 × 4.3 × 5.4 cm septal cystic, solid mass was detected on the left adnexal, and the solid components were enhanced.

A 52-year-old postmenopausal woman, G1P1, presented with abdominal pain and abdominal distension for more than 1 month was transferred to West China Second University Hospital. Physical examination revealed the bilateral adnexal thickness and tenderness. Her vulva, vagina, and cervix appeared normal and a Papanicolaou smear showed no abnormal cells. Tumor markers showed CA199:43.3 U/ml (<30.9), CA125:41.2 U/ml (<35), CEA:1.1 ng/ml (<2.5), AFP:4.2 ng/ml (<8.1). On transvaginal ultrasonography, a 7.1 × 4.3 × 5.4 cm septal cystic, solid mass was detected on the left adnexal and the endometrium was thin and clear. Abdominal computed tomography (CT) identified that the solid components of the mass were enhanced and left hydronephrosis with dilation of the proximal ureter and the lymph nodes of bilateral obturator regions, iliac vessels, and para-aortic were noticed (**Figure 1**).

**Figure 2:**
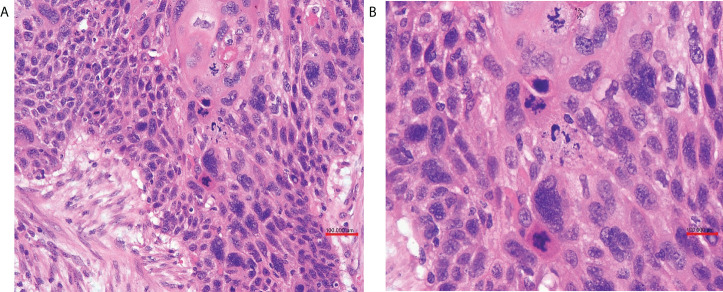
**(A)** The ovary was infiltrated by poorly differentiated squamous cell carcinoma (HE ×20 obj). **(B)** Tumor cells were pleomorphic, and mitotic figures were numerous (HE ×40 obj).

**Figure 3:**
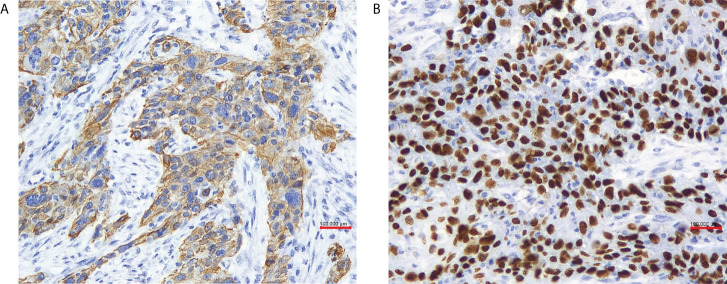
**(A)** In our case tumor cells were positive for cytokeratin 5/6 (x 20 obj), **(B)** P63 (x 20 obj).

The patient received laparoscopic evaluation. The pelvic mass was 8 cm in diameter origin from the left adnexa, densely aggregated with the surrounding sigmoid colon, omentum, and left pelvic peritoneal. A part of the left ureter was surrounded by the tumor in about 5-cm length. There was no ascitic fluid, and the right adnexa was normal. A frozen section of the left adnexal mass reported a poorly differentiated carcinoma of the ovary. A debulking surgery was performed, including total abdominal hysterectomy, bilateral salpingo-oophorectomy, pelvic and para-aortic lymph nodes dissection, omentectomy, appendectomy, and multiple peritoneal biopsies through a generous laparotomy, and there was no residual tumor. On grossly, the left adnexal mass section was grayish-white, solid, and crispy, and the fallopian tube and ovary were unremarkable. Her postoperative course was uneventful. Her postoperative pathology reported a poorly differentiated invasive SCC of the ovary without any other associated dermoid cysts, endometriosis, or Brenner tumors (**Figure 2**). The left fallopian tube, left pelvic peritoneal and lymph nodes, and the surface of the sigmoid colon showed tumor involved. The immunohistochemical assays of the ovarian mass revealed CK5/6+++、P63+++ (**Figure 3**)、P16+++、P53+、CK10/13++、UroIII-、CK7-、CK20-、Ki67+(>95%). Postoperative careful examination of head and neck, Sinus films have done. Repeat chest x-rays, Pelvic examination, and Pap smear showed no evidence that her malignant tumor was of non-ovarian origin. She had no symptoms of the esophagus or pancreas, or bladder, thus the final diagnosis was pure primary oSCC and staged as International Federation of Gynecology and Obstetrics (FIGO) stage IIIA.

The patient received adjuvant chemotherapy with carboplatin (450 mg) and paclitaxel (210 mg) at 4-week intervals for six courses. After completing six courses chemotherapy, abdominal CT revealed the thickening of the left pelvic peritoneal, and a solid, nodular mass occupied the left adnexal region. The mass involved the left iliac vessels, the left iliopsoas muscle, and the left lower ureter. Considering the possibility of tumor recurrence or metastasis, we combined the chemotherapy regimen with bevacizumab (400 mg) for two courses. However, 2 weeks after the treatment, the patient appeared bloody stool. Physical examination revealed a fixed mass with about 10 cm in diameter in the left pelvic cavity and an annular hollow about 1-cm long in the rectum. The patient chose to abandon the treatment and died of tumor progression and multiple organ failure 11 months after surgery.

## Discussion

Ovarian cancer clinical appearances are atypical, with 70–80% of patients diagnosed at advanced stages, and it is the most common cause of gynecological cancer death (15). Ovarian cancer is divided into diverse histologic subtypes, oSCC accounts for less than 1% of ovarian cancer, whereas most is epithelial ovarian cancer (EOC) (1, 2, 16). In most cases, the SCC of the ovary arises from dermoid cysts. Less frequently, SCC is associated with Brenner’s tumor, endometriosis, or endometrioid adenocarcinoma. Some are metastasis from other organs (e.g., uterine cervix, vagina, lung, esophagus, and neck) (1, 3). Pure primary oSCC is extremely rare. In 1964, Black and Benitez published the first article describing a pure primary SCC of the ovary, about 42 cases had been reported in the literature, and oSCC occurred at a mean age of 51 years old (range: 27–90 years) (1–14). Histogenesis of pure primary oSCC remains unclear. Some authors suggest that the coelomic epithelium is capable of squamous differentiation, or there are rare squamous cells of ovarian surface epithelium deteriorating into the tumor under the inflammatory stimulation (e.g., ovulation and medical injury) (1, 3, 4). The retrospective study of Jeong et al. revealed that about one-third of the pure primary oSCC patients had earlier or synchronous cervical intraepithelial lesions, and the majority were high-grade squamous intraepithelial lesions. Hence, it was supposed that the “field effect” caused by the common oncogenic agent HPV or contiguous spread along the female genital tract onto the ovary may be the main cause. However, the DNA or protein of HPV was not detected in these patients’ ovarian tumor tissues. Therefore, further research on the pure primary oSCC hypothesis is required (3, 4). The most common symptom is abdominal pain or a palpable abdominal mass, abdominal distension, and vaginal bleeding. Some may complain of metastasis or local extension symptoms such as cough and rectal bleeding. Often found incidentally in intraoperative or postoperative (4).

Because SCC is the common histologic type of malignancy in the cervix, vulva, vagina, lung, head, and neck. We are of the view that thorough evaluation, including physical examination, cytologic tests if applicable, and/or imaging of these anatomic sites, is mandatory to make the primary diagnosis. Especially with the immunohistochemistry (IHC) site-specific markers (3). The oSCC should also display strong positive squamous markers: CK34bE12 or CK5/6、p63. All the three germ layer elements surrounding the SCC lesion indicated that the dermoid cysts were malignant. Iron stains to highlight the presence of hemorrhage and hemosiderin support the diagnosis of squamous carcinoma arising from endometriosis. Vimentin, CK7, ER, p16, and p53 may help to distinguish squamous metaplasia in an endometrioid adenocarcinoma (1, 5). Additionally, positive HPV and p16 may indicate the cervical primary. The distant organs (e.g., lung and breast) metastatic cancer shows CK-7 positivity (3). In this case, the patient showed strong positive for CK5/6 and p63, whereas no reactivity with CK7. On pathology, no other components were identified, including cystic teratoma, Brenner tumor, endometriosis, or mucinous cystadenoma. Postoperative careful examinations of the head and neck, Sinus films, repeat chest x-rays, pelvic examination, and Pap smear showed no dysplasia or neoplasia. The final diagnosis of pure primary oSCC was convinced.

oSCC is rare but has poor prognosis; Cheng, based on the SEER database, selected patients with oSCC and serous carcinoma (SC) with 1:2 matching analysis and revealed that the median survival time of oSCC was 21 months. The 1-, 2-, and 5-year OS rates of oSCC were 61.6, 49.2, and 44.9%, respectively. The prognosis of SCC was significantly worse than that of patients with SC (6). The outcome of oSCC is strongly determined by the stage at diagnosis. The 5-year survival rate of stage I is 76%, whereas the 5-year survival rates of advanced stages are 34, 21, and 0% for stages II, III, and IV, respectively (4, 7). Only a few cases of advance-staged pure primary SCC have been illustrated in previous reports (**Table 1**) (4, 7, 8, 10, 11). The FIGO stages III–IV patients’ 1-year survival rate was 29.4%, the 2-year survival rate was 17.6%, and only one patient survived more than 5 years (4, 6–11). oSCC has locally invasive characteristics; the tumors were very invasive or adherent to the adjacent uterus, pelvic, and colonic peritoneum; and surgery is the crucial treatment. Research indicates that debulking with lymphadenectomy might have survival benefits (3, 4, 12). Older age at diagnosis, larger tumor size, and bilateral tumor are factors for poor survival of oSCC (4, 6).

**Table 1:** Summary of literature-based papers referring to advanced-stage pure primary oSCC.

Authors	Age	Stage	Surgical Treatment	Adjuvant therapy	Follow-up
Ben-Baruch et al	65	III	TD	Cyc/Adriamycin/Cis	DOD 6 mo
Kashimura et al	42	III	LSO	RT	DOD 8 mo
Radhi et al	64	IV	TD	No	DOD 9days
Pins et al.	55	IIIB	HBSO	Chemo	DOD 2 mo
Pins et al.	52	IIIC	Ovarian, omental biopsy	NA	NA
Pins et al.	46	IIIC	Ovarian, omental biopsy	NA	NA
Pins et al.	27	IIIC	HBSO	Chemo	DOD 2 mo
Pins et al.	70	IIIC	HBSO	Chemo	DOD 5 mo
Pins et al.	73	IV	LSO	RT	DOD 1 mo
Eltabbakh GH et al.	31	IV	TD	Pac/Cis×12	ANED 12 mo
Yukiharu et al.	56	IIIC	TD	1^st^ line: Pac/Car×5 2^nd^line: Cis/bleomycin/mitomycin C/vincristin	DOD 11 mo
Chien et al	63	IV	TD	Pac/Cis×6	DOD 7 mo
Amjad and Pal	31	IIIC	HBSO, TO, BR	Cis/Etoposide×3	DOD 3 mo
Park et al.	48	IV	TD	Pac/Platinum×3	AWD 9 mo
Park and Bae	46	IV	HBSO, BR	1^st^ line: Pac/Car×6 2^nd^ line: Top/Cis×3 3^rd^ line I/E×3	DOD 12 mo
Sharma et al.	66	IIIC	HBSO, I-O, Removal of right parietal wall mass	Cis+ RT	DOD 2 mo
Tang et al.	45	IIIC	Interval TD	Pac/Car×6	ANED 42 mo
Shrivastava et al	30	IIIC	TD	Pac/Cis×7	DOD 12 mo
Hiroyuki et al	71	IIIC	LO, RSO, BR	Pac/Car×5, CPT-11/Cis×6	ANED 60 mo
Koufipoulos et al	55	IIIC	HBSO	1^st^ line: Pac/Cis×1 Pac/Car×5 2^nd^ line: Gemcitabine×1	DOD 9 mo

TD, optimal tumor debulking; L/R-SO, left/right salpingo-oophorectomy; HBSO, hysterectomy and bilateral salpingo-oophorectomy; TO, total omentectomy; I-O, infracolic omentectomy; B, bowel resection; LO, left oophorectomy; Pac, paclitaxel; Cis, cisplatin; Car, carboplatin; Top, topotecan; CPT-11, Irinotecan; Cyc, cyclophosphamide; I/E, ifosfamide/etoposide; NA, not available; RT, radiotherapy, ANED, alive no evidence of disease, AWD, alive with disease; DO, died of disease.

Pure primary oSCC is extremely rare; the adjuvant treatment has not been well established. Because this tumor is regarded as EOC, the adjuvant chemotherapy with paclitaxel and platinum should be suggested (3, 4, 6). Other drugs such as irinotecan, topotecan, etoposide, ifosfamide, and so forth have been used as second- or third-line treatment (11, 13). Paclitaxel and platinum-based regimens have showed good results (8, 10, 11). On the contrary, not-so-good responses also have been reported (7, 13, 14). Given that SCC is a radiosensitive tumor, radiation and concurrent chemotherapy following aggressive cytoreduction be of benefit for oSCC arising from teratomas in some case series. The attempt of pains to treat a pure primary oSCC FIGO stage IIB patient with chemoradiotherapy achieved 60 months alive with no evidence of disease after surgery. However, the most advanced pure primary oSCC showed no effectiveness to radiotherapy (9, 14).

In addition, treatments for other organs SCC may offer a clue for the treatment of pure primary oSCC. EGFR is overexpressed in cervical SCC. A phase II study of EGFR inhibition combined with radiotherapy and cisplatin in the treatment of locally advanced cervical cancer has shown a complete response rate of 94.4%. A patient with metastatic oSCC was treated with a combination of platinum, cetuximab, and radiotherapy, and the patient achieved an impressive 5-year disease-free survival (1). Vascular endothelial growth factor (VEGF) involves in tumor angiogenesis. Recent study demonstrated that the adjuvant of the humanized anti-VEGF monoclonal antibody combined with chemotherapy for treatment in recurrent, persistent, or metastatic cervical cancer had achieved a 3.7-month improvement in overall survival (1, 4). In our case, after six courses of carboplatin combined with paclitaxel chemotherapy, the imaging results suggested tumor recurrence or metastasis, so bevacizumab was added to carboplatin combined with paclitaxel two more times. Unfortunately, due to the rapid progression of the disease progression, the patient died 11 months after surgery. Its effectiveness in the treatment of pure primary oSCC has not been observed. The chemotherapy regimens or their dosages may be ineffective to our patients, and more clinical investigations are needed.

## Conclusion

The pure primary oSCC is extremely rare and most reported in case reports. The effective treatment has been not established. At present, debulking surgery followed by chemotherapy paclitaxel combined with platinum is recommended. However, due to the variable prognosis, the adjuvant therapy is still controversial. With the accumulation of more cases, drawing lessons from the existing treatment of primary SCC in other organs and other types of EOC, as well as the clarification of its pathological mechanism, a breakthrough in the treatment of pure primary oSCC will be achieved.

## Data availability statement

The raw data supporting the conclusions of this article will be made available by the authors, without undue reservation.

## Ethics statement

Written informed consent was obtained from the individual(s) for the publication of any potentially identifiable images or data included in this article.

## Author contributions

YL: manuscript writing. CB: protocol development, manuscript revision, and final approval of the version to be published. All authors listed have made a substantial, direct, and intellectual contribution to the work and approved it for publication.

## Conflict of interest

The authors declare that the research was conducted in the absence of any commercial or financial relationships that could be construed as a potential conflict of interest.

## Publisher’s note

All claims expressed in this article are solely those of the authors and do not necessarily represent those of their affiliated organizations, or those of the publisher, the editors and the reviewers. Any product that may be evaluated in this article, or claim that may be made by its manufacturer, is not guaranteed or endorsed by the publisher.
